# The Influence of Wireless Self-Monitoring Program on the Relationship Between Patient Activation and Health Behaviors, Medication Adherence, and Blood Pressure Levels in Hypertensive Patients: A Substudy of a Randomized Controlled Trial

**DOI:** 10.2196/jmir.5429

**Published:** 2016-06-22

**Authors:** Ju Young Kim, Nathan. E Wineinger, Steven. R Steinhubl

**Affiliations:** ^1^ Digital medicine Scripps Translational Science Institute La Jolla, CA United States; ^2^ Department of Family Medicine Seoul National University Bundang Hospital Seongnam Republic Of Korea

**Keywords:** patient participation, blood pressure self-monitoring, wireless technology, telemedicine, health behavior, medication adherence

## Abstract

**Background:**

Active engagement in the management of hypertension is important in improving self-management behaviors and clinical outcomes. Mobile phone technology using wireless monitoring tools are now widely available to help individuals monitor their blood pressure, but little is known about the conditions under which such technology can effect positive behavior changes or clinical outcomes.

**Objective:**

To study the influence of wireless self-monitoring program and patient activation measures on health behaviors, medication adherence, and blood pressure levels as well as control of blood pressure in hypertensive patients.

**Methods:**

We examined a subset of 95 hypertensive participants from a 6-month randomized controlled trial designed to determine the utility of a wireless self-monitoring program (n=52 monitoring program, n=43 control), which consisted of a blood pressure monitoring device connected with a mobile phone, reminders for self-monitoring, a Web-based disease management program, and a mobile app for monitoring and education, compared with the control group receiving a standard disease management program. Study participants provided measures of patient activation, health behaviors including smoking, drinking, and exercise, medication adherence, and blood pressure levels. We assessed the influence of wireless self-monitoring as a moderator of the relationship between patient activation and health behaviors, medication adherence, and control of blood pressure.

**Results:**

Improvements in patient activation were associated with improvements in cigarette smoking (beta=−0.46, P<.001) and blood pressure control (beta=0.04, P=.02). This relationship was further strengthened in reducing cigarettes (beta=−0.60, P<.001), alcohol drinking (beta=−0.26, P=.01), and systolic (beta=−0.27, P=.02) and diastolic blood pressure (beta=−0.34, P=.007) at 6 months among individuals participating in the wireless self-monitoring program. No differences were observed with respect to medication adherence.

**Conclusions:**

Participation in a wireless self-monitoring program provides individuals motivated to improve their health management with an added benefit above and beyond that of motivation alone. Hypertensive individuals eager to change health behaviors are excellent candidates for mobile health self-monitoring..

**Trial Registration:**

ClinicalTrials.gov NCT01975428, https://clinicaltrials.gov/ct2/show/NCT01975428 (Archived by WebCite at http://www.webcitation.org/6iSO5OgOG)

## Introduction

Hypertension is the single greatest attributable risk factor for death and disease burden worldwide from noncommunicable disease [1]. Management of blood pressure has been shown to reduce the risk of stroke, heart attack, heart failure, and cardiovascular death [2,3]. However, less than half of young adults and two-thirds of older adults with treatment-eligible hypertension meet blood pressure goals [4].

The success of long-term effective control of blood pressure largely depends on patient self-management, where the individual plays a central role in their decisions and behaviors regarding management of this chronic condition. Self-management skills in chronic conditions include medication adherence, dietary compliance, self-monitoring of key parameters such as blood pressure, and coping skills to better manage anger or frustration that comes from living with disease [5,6]. A variety of tools to improve self-management have been evaluated as strategies for improving the treatment of a number of chronic conditions, most by emphasizing patient education and by creating a more active role of the patient in their health [7]. A critical part in self-management is active, engaged patients.

The Patient Activation Measure (PAM) is one of the tools for measuring an individual’s skills, confidence, and knowledge in managing his or her own health [8,9]. PAM scores can be categorized into 4 levels: (1) the patient as a passive recipient of health care, (2) interested but lacking knowledge and confidence to act, (3) taking actions but lacking confidence and skills to support new behaviors, and (4) adoption of new behaviors but may not necessarily sustain them under crisis or stress. 

Higher PAM scores have shown strong relationships with better performance of self-management behaviors, higher medication adherence, and higher quality-of-life score in patients with chronic condition [10]; clinical outcomes such as hemoglobin A_1c_levels in diabetic patients [11]; and lower health care utilization, emergency room visits, and admissions [12]. PAM score has also been associated with changes in health behaviors, safety, cancer risk, stress, and mental health [13].

With the development of mobile medical technologies, tools to enhance self-management that combine home monitoring with near real-time bidirectional communication and feedback are becoming more widely available through mobile phones and wireless hubs [14]. The greatest strength of mobile health (mHealth) for patients may be in monitoring their disease [15]. Mobile health devices that measure physiological metrics allow individuals to track their health condition, diagnose health problems, or identify early warning signals without the direct need of a health care provider. A recent randomized clinical trial that used home blood pressure telemonitoring combined with automated self-care support using mobile phone found that mean daytime ambulatory systolic blood pressure decreased significantly by more than 9 mm Hg in the intervention group [16]. Several clinical trials have also proven that self-monitoring of blood pressure, when coupled with health care provider feedback, can result in improved blood pressure control [17,18].

However, the mechanism of improved blood pressure control in individuals utilizing blood pressure self-monitoring is still not well understood. According to a meta-analysis of blood pressure self-monitoring on medication adherence and lifestyle factors from 28 trials with 7021 participants, monitoring was shown to have a small but significant effect on medication adherence but an inconclusive result related to diet and physical activities [19]. One other study found that using a monitoring system over 3 months improved PAM scores, self-care, and quality of life in elderly patients with heart failure [20]. However, another study found no relationship between PAM score and blood pressure in patients with uncontrolled hypertension receiving a comprehensive home care and monitoring system [21].

We originally performed the prospective, randomized controlled trial of the effect of wireless self-monitoring program on health care utilization in a heterogeneous group of patients with hypertension, type 1 or type 2 diabetes mellitus, or cardiac arrhythmia [22]. This study sought to determine whether wireless self-monitoring and PAM could be an effective way for improving health outcomes in hypertensive patients. Importantly, if wireless blood pressure self-monitoring programs can improve patient activation and downstream health behaviors, medication adherence, and blood pressure control, then strategies for such programs can be optimized to influence patient activation.

However, to date there have been limited studies of PAM, its modifiability through mHealth blood pressure self-monitoring, and their effects on behavioral factors, medication adherence, and blood pressure levels in a hypertensive population. Our study aimed to evaluate the interaction between wireless self-monitoring program and PAM in positive behavior changes, medication adherence, and control of blood pressure among hypertensive patients.

## Methods

### Design and Setting

This study was a secondary analysis of a prospective, randomized controlled, 2-group, pre-post intervention trial on the 6-month effectiveness of mobile phone–based self-monitoring program of chronic conditions on health care utilization [22]. The trial took place between July 2013 and December 2014, was approved by the Scripps Institutional Review Board (approval no: IRB-12-6019), and registered (trial registration: ClinicalTrials.gov identifier NCT01975428)].

In brief, this study was a collaboration between Scripps Translational Science Institute, Scripps Health, HealthyCircles by Qualcomm Life, and HealthComp, the third-party administrator for Scripps Health responsible for processing all health care insurance claims for employees and dependents. Study participants in the primary trial were drawn from a pool of eligible employees and dependents insured by Scripps Health in 2012 and identified to have a *Current Procedural Terminology* (CPT) code related to one or more of the following diagnoses: hypertension, diabetes, or cardiac arrhythmia according to a medical claims database. The 3998 eligible individuals were identified according to inclusion criteria of being able to attend visits at a Scripps facility, being able to access the Internet, participating in HealthComp disease management program, and being willing to use wireless devices, iPhone, and the HealthyCircles Platform. They were also identified as the highest 25% in terms of their 2012 medical billing data to account for the highest health care spending and resource utilization. Individuals were recruited through an emailed invitation letter, followed up with a telephone call. If individuals expressed interest in the study, they were provided with a link to a Web-based informed consent form through email. After consenting to the study, study participants completed a Web-based survey that included demographic, health, and technology-related questions. Afterward, participants were randomized to control or intervention and brought in for an enrollment visit with an unblinded research coordinator, which they repeated at the conclusion of their 6-month study enrollment at a Scripps facility. Participants were blind to their assigned group before enrollment. All participants were randomized (ratio 1:1) to either intervention group, receiving wireless monitoring program plus disease management, or control group, receiving standard disease management program.

**Figure 1:**
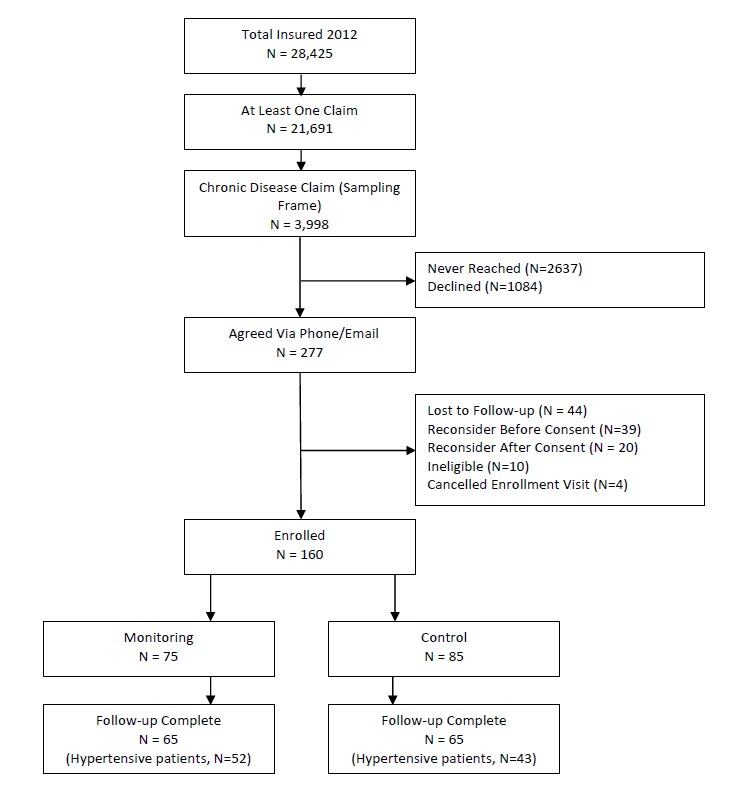
Study enrollment flowchart.

Our study focused only on those enrolled in the original study with a diagnosis of hypertension, prescribed at least one or more antihypertensive medications, and completed their 6-month follow-up study visit. The flow of study participants and subset of participants in this study are presented in Figure 1.

### Wireless Self-Monitoring and Control Groups

During the study enrollment visit, all study participants completed the baseline survey if not already completed online and underwent height, weight, and blood pressure measurements, as well as a 12-lead electrocardiogram and fasting blood glucose test in the case of hypertensive individuals with a history of cardiac arrhythmia and diabetes, respectively.

**Figure 2:**
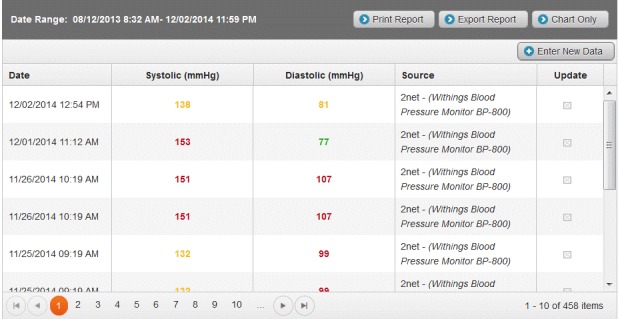
Screenshot of HealthyCircles online portal in wireless monitoring program.

**Figure 3:**
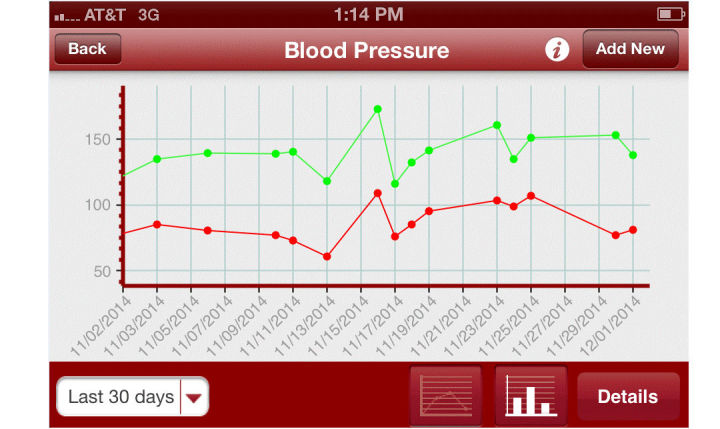
Screenshot of self-monitoring blood pressure data on mobile phone.

Participants randomized to the monitoring group were provided with a study monitoring device according to their condition. Those enrolled with a diagnosis of hypertension received a Withings Blood Pressure Monitor. Participants in the monitoring group also received an iPhone with corresponding apps and were enrolled in the HealthyCircles Platform—an online disease management program featuring educational materials, consumer portals, and a dashboard to link with their families, caregivers, and health care professionals. HealthComp is the third-party administrator for Scripps Health, and HealthComp nursing staff had access to the HealthyCircles care management dashboard that showed the participant’s device monitoring results and trends over time. Readings recorded on the devices were wirelessly uploaded to the HealthyCircles account and were accessible to the patient as well as the HealthComp nurses via an iPhone or computer. Also included in the management platform were reminders for monitoring, information about the disease condition, and general health behavior recommendations. Hypertensive participants in the monitoring group were trained on how to use their mobile phone, the HealthyCircles mobile app, portal, and their device. They were encouraged to use the device 3 times a week, taking 2 measurements per day, with the first in the morning. If their monitoring frequency fell below 3 times per week for 2 consecutive weeks, participants received an email on their HealthyCircles Platform reminding them of the monitoring schedule. If participants were recommended more frequent monitoring by their physician than that asked through the study, they were encouraged to follow the physician’s instructions. Figures 2 and 3 are screenshots of the HealthyCircles online portal and mobile app.

All participants, including participants randomized to control group, were enrolled in the HealthComp disease management program, and HealthComp nursing staff could reach out to all participants for the purpose of relaying medical education and wellness information with regard to disease prevention and chronic disease management.

### Outcome Measurements

Three health behaviors were assessed at both baseline and the 6-month follow-up Web-based survey: frequency of the use of alcohol, smoking, and exercise. Alcohol frequency was assessed by one survey item asking, “In the *past 6 months*, how many alcoholic drinks per week do you/did you have? (if you are a current or previous drinker)?” and the answer was coded as one of following categories: never, monthly or less, 2-4 times a month, 2-3 times a week, and 4 or more times a week. Smoking frequency was assessed by one survey item asking, “In the *past 6 months*, how many cigarettes/cigars per day do you/did you smoke? (if you are a current or previous user of tobacco)” and the answer was coded as one of the following categories: never, 10 or fewer, 11 to 20, 21 to 30, and 31 or more cigarettes. Finally, exercise frequency was assessed using the validated Godin Leisure-Time Exercise Questionnaire (GLTEQ) [23]. The GLTEQ consisted of average frequency of mild (minimal effort), moderate (not exhausting), and strenuous (producing rapid heartbeats) activities undertaken for more than 15 minutes during a typical week. Medication adherence was likewise assessed using a validated 8-item self-reported questionnaire [24]. Systolic and diastolic blood pressure outcomes were based on those measured by a research nurse using the Withings Blood Pressure Monitor at the baseline and study completion visits. Finally, individuals were classified as either having or not having adequate blood pressure control based on recommendations from the Eighth Joint National Committee (JNC 8) [25].

### Determinants

The primary independent variables of interest were (1) assignment to the wireless self-monitoring or control group and (2) the relative change in the PAM score from baseline to the end of the study. Our primary hypothesis examined the moderator effect of this monitoring group assignment on the relative change in PAM on the outcome measures. That is, we jointly modeled the main effects of wireless self-monitoring and relative change in PAM, and the interaction between these variables. There was a problem regarding the database where a subset of Withings measurements could not be accurately determined, so self-monitoring was simply treated as a dichotomous variable.

Meanwhile, PAM was derived from an interval level, unidimensional, Guttman-like measure questionnaire consisting of 13 items. Each item has 4 possible responses ranging from “disagree strongly” (coded as 1) to “agree strongly” (coded as 4). An overall PAM score can be calculated by summing the coded responses. Overall PAM scores are often used to place individuals into 1 of 4 levels based on thresholds. However, as we were interested in changes in patient activation from baseline, we retained the overall scores and calculated the relative change in PAM as the difference in activation score between baseline and follow-up divided by baseline activation score.

### Potential Confounders

To account for potential confounding factors, several sociodemographic factors were modeled as potential covariates: age, sex, education level and income level, baseline blood pressure, the number and types of medications (ie, lipid-lowering drug, glucose-lowering drug, insulin, use of antidepressant medication, and anxiolytic medication), and the frequency with which an individual visited a physician in the 6 months before starting the trial.

### Statistical Analysis

Because the original trial aimed to evaluate health care resource utilization as measured by health insurance claims and visits to the hospital including office visits, emergency room visits, inpatient stays, and all visits during the study periods, sample size was determined to be powered (a priori) to detect a 1–office visit difference between the control and the monitoring group (assuming a standard deviation of 2 office visits). Univariate analyses were performed on each of the variables assessed. Multivariable regression was performed in a stepwise approach to determine the effects of self-monitoring, relative changes in PAM, and their interaction on behavior changes, medication adherence, blood pressure, and blood pressure control. In total, 3 models were constructed: (1) covariates only, (2) covariates and the main effects of self-monitoring and relative changes in PAM, and (3) covariates, main effects, and their interaction. Covariates were selected based on marginal associations (*P*<.10) with the outcomes. All data analyses were performed using R software, version 3.2.0 [26].

## Results

Our analysis focused on those study participants with a hypertension history who were prescribed antihypertensive medication according to claims data, completed both surveys, and provided blood pressure measurements at both study visits (n=95). This includes 52 participants assigned to the monitoring group and 43 participants assigned to the control group.

Table 1 lists the baseline characteristics of study participants. The mean age of participants was 57.6 years, with most participants being female (65/95, 68%), Caucasian (76/95, 80%), with most completing college (78/95, 82%), and generally in the middle-income bracket. The majority of participants were also nonsmokers (68/95, 72%) with about half drinking less than once per week (52/95, 55%) and being an active exerciser by GLTEQ category (41/95, 43%). In terms of clinical factors, in addition to a recent history of hypertension, around one-third of the study participants had a recent history of diabetes and/or cardiac arrhythmia. The mean number of antihypertensive medications per individual at baseline was 2.0, with 26% (25/95) taking an oral glucose-lowering medication, 54% (51/95) took lipid-lowering medication, and 34% (32/95) took antidepressant medication. Consistent with the randomization, there were no differences in baseline characteristics between the control and self-monitoring groups that could be explained outside of chance.

Summary statistics of independent and dependent variables of interest are presented in Table 2. The average baseline PAM score across all participants was 78.0 and was not significantly different from the end of study scores (mean 76.0, *P*=.34). Meanwhile, the average number of cigarettes smoked per day across self-identified smokers significantly decreased from 16.7 to 1.9, and the percentage of nonsmokers increased from 72% to 96%. However, the average frequency of alcohol use (baseline: 5.5 drinks per month, end of study: 5.7; *P*=.94) and exercise group by GLTEQ categories (baseline: 36.8, end of study: 40.5; *P*=.17) did not change over the course of the study. Baseline Morisky Medication Adherence Scale (MAS) scores also did not change over the study period (baseline: 6.5, end of study: 6.6; *P*=.46). However, the average systolic and diastolic blood pressure levels decreased across all study participants over the study period (baseline: 140.6/89.4 mm Hg; end of study: 136.5/83.9 mm Hg). Furthermore, according to JNC 8 guidelines, the frequency of achieved blood pressure control increased from 45% to 59%.

Results of the multivariable models examining the relationship with PAM, wireless self-monitoring, and their interaction with the outcome variables of interest are presented in Table 3. The interaction between wireless self-monitoring and positive changes of PAM was a significant contributor to cigarette smoking (beta = −0.60, *P*<.001), alcohol drinking (beta = −0.26, *P=*.01), systolic blood pressure (beta = −0.27, *P=*.02), and diastolic blood pressure (beta = −0.34, *P=*.007) at 6 months. In all these cases, the wireless self-monitoring group had greater decreases in these outcome variables than the control group. This interaction was not a significant predictor of change in exercise per week, Morisky MAS, or achieved blood pressure control—although the main effects of both self-monitoring and changes in PAM were conditionally associated with achieved blood pressure control.

In order to further dissect these interactions, we evaluated the relationship between relative changes in PAM and number of cigarettes smoked per day, frequency of alcohol drinking, and systolic and diastolic blood pressure in the self-monitoring group and control group separately. We observed that increases in PAM scores were significantly associated with a decrease in smoking (beta = −0.63, *P*<.001), frequency of alcohol drinking (beta = −0.22, *P*=.04), systolic blood pressure (beta = −0.47, *P*<.001), and diastolic blood pressure (beta = −0.42, *P*<.001) in the monitoring group. However, no association was observed in the control group: smoking (beta = −0.11, *P*=.55), frequency of alcohol drinking (beta = 0.13, *P*=.48), systolic blood pressure (beta = 0.10, *P*=.46), or diastolic blood pressure (beta = −0.01, *P*=.93).

We also evaluated the temporal trends of frequency in self-monitoring of blood pressure as well as mean systolic and diastolic blood pressure levels in a subset of individuals (33/52) whose self-monitoring data were available during the total study period (Multimedia Appendix 1).

After 12 weeks, about 70% (23/33) had monitored their blood pressure regularly at a recommended frequency (median 6 per week, interquartile range [IQR] 5-9), and at the end of the study period, 55% (18/33) had still participated in the self-monitoring program with a median frequency of 6 per week (IQR 5-7; Multimedia Appendix 1). Baseline systolic and diastolic blood pressure levels in self-monitoring database were 133.6 mm Hg and 82.9 mm Hg, respectively. At the end of the study, systolic blood pressure was 129.7 mm Hg and diastolic was 78.4 mm Hg, showing decreased pattern over time but not a clinically meaningful decrease in terms of controlled hypertension.

**Table 1:** Baseline characteristics of hypertensive patients (n=95).

Characteristics		Monitoring (n=52)	Control (n=43)	Total	*P* value
**Sociodemographic factors**					
Age in years, mean (SD)		57.5 (8.6)	57.7 (8.7)	57.6 (8.6)	.89
Sex, n (%)					
	Female	38 (73)	27 (63)	65 (68)	.28
Ethnicity, n (%)					.07
	Caucasian	43 (83)	33 (77)	76 (80)	
	African American	4 (8)	2 (5)	6 (6)	
	Hispanic	4 (8)	1 (2)	5 (5)	
	Asian	1 (2)	4 (9)	5 (5)	
Married, n (%)		29 (56)	30 (70)	59 (62)	.40
Level of education, n (%)					
	≤12 years	6 (12)	11(26)	17 (18)	.09
	Complete college	22 (42)	20 (47)	42 (44)	
	More than college	24 (46)	12 (28)	36 (38)	
Income^a^, n (%)					
	<50K	5 (10)	5 (12)	10 (11)	.81
	50-149K	35 (67)	32 (74)	67 (70)	
	150-249K	10 (19)	1 (2)	11 (12)	
	≥250K 2 (4)	5 (12)	7 (8)		
Alcohol, n (%)					
	None	7 (13)	11(26)	18 (19)	.22
	Less than 1/week	28 (54)	24 (56)	52 (55)	
Smoking, n (%)					
	None	32 (62)	36 (84)	68 (72)	.07
Exercise, n (%)					
	Active	23 (44)	18 (42)	41 (43)	.82
**Clinical factors**					
Number of antihypertensive medication		1.9 (1.0)	2.1 (1.0)	2.0 (1.0)	.38
Self-reported frequency of physician clinic visits in the past 6 months^a^		3.0 (4.4)	2.6 (1.7)	2.9(2.5)	.56
Comorbidity, n (%)					
	Type 1 Diabetes Mellitus	7 (13)	4 (9)	11 (12)	.53
	Type 2 Diabetes Mellitus	4 (8)	6 (14)	10 (11)	.32
	Arrhythmia	4 (8)	8 (19)	12 (13)	.11
Comedication, n (%)					
	Insulin	7 (13)	5 (12)	12 (13)	.79
	Diabetic medication	12 (23)	13 (30)	25 (26)	.43
	Lipid-lowering medication	25 (48)	26 (60)	51 (54)	.23
	Antidepressant	18 (35)	14 (33)	32 (34)	.83
	Anxiolytics	5 (10)	6 (14)	11 (12)	.51

^a^Variable with significant difference between monitoring group and control group.

**Table 2:** Patient activationmeasure, health behaviors, medication adherence, and blood pressure measures.

Characteristics		Baseline, mean (SD)	After 6 months, mean (SD)	*P* value
**Predictors**				
Patient Activation Measure				
	Self-monitoring group	82.9 (13.8)	79.4 (21.9)	.19
	Control group	72.0 (16.4)	72.1 (18.7)	.96
**Outcome measurements**				
**Health behaviors**				
Cigarettes per day among current smokers				
	Self-monitoring group	16.5 (9.3)	2.6 (7.3)	<.001
	Control group	17.1 (7.6)	0.3 (1.6)	.03
Frequency of drinking per month among drinkers				
	Self-monitoring group	7.2 (8.0)	7.6 (7.9)	.66
	Control group	6.2 (7.7)	5.8 (6.8)	.69
Exercise units per week				
	Self-monitoring group	37.8 (26.1)	39.8 (23.8)	.58
	Control group	35.6 (26.6)	41.3 (29.5)	.18
**Morisky MAS** ^a^				
	Self-monitoring group	6.6 (1.4)	6.7 (1.4)	.79
	Control group	6.3 (1.4)	6.5 (1.5)	.47
**Achieved blood pressure control, n (%)**				
	Self-monitoring group	29 (56)	34 (65)	.23
	Control group	14 (33)	22 (51)	.001
**Systolic blood pressure**				
	Self-monitoring group	136.1 (15.2)	133.4 (12.9)	.28
	Control group	145.9 (19.5)	140.2 (18.4)	.06
**Diastolic blood pressure**				
	Self-monitoring group	86.3 (12.8)	82.8 (11.2)	.06
	Control group	93.1 (14.1)	85.3 (12.1)	.001

^a^Morisky MAS: Morisky Medication Adherence Scale 8 item.

**Table 3:** Multivariable regression model parameters estimating the effect of changes in PAM, wireless self-monitoring, and their interaction on the changes in health behaviors, medication adherence, and blood pressure at 6 months in all hypertensive patients (standardized regression coefficients and R^2^ change with significance).

Model	Smoking (cigarettes/day)	Alcohol (frequency/month)	Exercise (unit/week)	Morisky MAS^a^	Systolic BP^b^	Diastolic BP	Achieved BP control
**Model 1**							
	R^2^=0.15	R^2^=0.60	R^2^=0.39	R^2^=0.47	R^2^=0.29	R^2^=0.20	R^2^=0.36
Covariates							
**Model 2**							
	Δ R^2^= 0.21	Δ R^2^= 0.02	Δ R^2^= 0.00	Δ R^2^= 0.00	Δ R^2^= 0.02	Δ R^2^= 0.03	Δ R^2^= 0.07
Δ PAM^c^	−0.46; *P*<.001	−0.05; *P*=.56	0.01; *P*=.89	0.02; *P*=.85	−0.12; *P*=.25	−0.25; *P*=.06	0.04; *P*=.02
Self-monitoring	0.04; *P*=.84	0.10; *P*=.19	−0.03; *P*=.78	−0.03; *P*=.60	−0.18; *P*=.07 −0.06; *P*=.50	0.6; *P*=.03	
**Model 3**							
	Δ R^2^= 0.21	Δ R^2^= 0.03	Δ R^2^= 0.02	Δ R^2^= 0.00	Δ R^2^= 0.05	Δ R^2^= 0.06	Δ R^2^= 0.00
PAM*self-monitoring	−0.60; *P*<.001	−0.26; *P*=.01	0.15; *P*=.13	0.07; *P*=.48	−0.27; *P*=.02	−0.34; *P*=.007	0.01; *P*=.12

^a^Morisky MAS: Morisky Medication Adherence Scale 8 item.

^b^BP: blood pressure; PAM: Patient Activation Measure (13 items).

^c^Δ PAM**:** difference in PAM in 6 months/baseline PAM.

## Discussion

### Principal Findings

One goal of many chronic disease management programs is to adopt patient activation enhancing strategies in management of chronic disease. Such programs are generally designed to enhance problem solving, decision making, and taking actions [27]. These characteristics are common behavioral components of mobile self-monitoring and health intervention apps [28]. This study explored the moderating effects of wireless self-monitoring on relationships between changes of PAM score and changes of health behaviors, medication adherence, and blood pressure in individuals with hypertension over a 6-month period. The wireless self-monitoring program consisted of mobile phone–based self-monitoring with an online Web portal used for social support, education, reminders, and to track recordings, because this program was designed to leverage collaborations with device manufacturers, a connected health leader, health care provider, as well as employee wellness program.

We discovered that changes in PAM were associated decreases in the number of cigarettes smoked, alcohol consumed, and blood pressure in the self-monitoring group alone. This suggests wireless self-monitoring program may reinforce changes of PAM by enhancing the ability to make healthy decisions and to manage blood pressure.

To date there have been few studies that have explored the relationship between wireless self-monitoring and changes of PAM in chronic care. One study examining African Americans with uncontrolled hypertension showed that blood pressure self-monitoring did not have any effect on changes of PAM [21]. Another study suggested that online social networks had favorable effects on changes of PAM in individuals with lower activation scores [29]. However, the study did not have the component of online self-monitoring in chronic disease population. Among 95 of our study’s participants, 72 (76%) would be placed in the highest level of patient activation while 86 (91%) of participants were in the patient activation level 3 or higher. Thus, our study results support the finding that wireless self-monitoring can strengthen improved changes in PAM, even in the highest levels of patient activation [13].

We further discovered that changes in PAM were conditionally associated with changes in health behaviors and blood pressure control. These results support the finding that an increase in PAM score may improve health outcomes and potentially decrease long-term medical costs [30]. Importantly, this benefit appeared to be exclusive to individuals in the wireless self-monitoring group, suggesting that future strategies should strongly consider implementing mHealth in chronic disease management.

Despite this relationship between changes in PAM and blood pressure control, changes in PAM were not significantly associated with medication adherence. We observed a median Morisky MAS score of 7.0, which indicated relatively medium to high adherence. Our study participants consisted of Scripps Health hospital employees or their families. Thus, participants were likely to have higher health literacy or more availability of health personnel resources than the general population, which could possibly lead to better control of their condition. Furthermore, study participants generally had well-controlled hypertension, because the study participants took on average 2 antihypertensive medications and had a baseline blood pressure of 140.6/89.4 mm Hg, indicating generally mild to moderate hypertension. Finally, one previous study showed that uncontrolled depression was a barrier to antihypertensive medication adherence and was negatively associated with PAM [31]. Although one-third of our study’s participants had been prescribed antidepressant medication, there is a high possibility that depression symptoms were also well controlled in our study. Further studies should examine these relationships in higher-risk populations.

The mechanism by which a wireless self-monitoring program potentiates the effect of changes in PAM on health behaviors and outcomes in hypertensive participants remains unknown. Although many studies have shown that patient activation level is associated with improved health behaviors and clinical outcomes [11,13,30,32], studies regarding effective intervention to enhance patient activation are limited. One prior study showed that remote monitoring with alert system increased activation levels in 21 patients with heart failure over 3 months [33].

In this study, the wireless self-monitoring program had integrative multiple components including online portal with social support from their family or friends, reminder for monitoring blood pressure, and education about the disease, as well as recommendation for positive behavior change for better outcomes. A study showed that Internet-based behavior intervention grounded in theory proved to be effective [34]. Among several proven theories, external supports such as personalized email or reminders to engage users in the intervention and providing peer support may improve adherence to specific intervention and outcomes in improving type 2 diabetes [35]. The availability of personal health-related data to individuals enrolled in the wireless self-monitoring group could have reinforced these changes. However, there is a great need for proven mHealth evidences in preventing cardiovascular disease [36].

In this study, Withings blood pressure monitoring device was used to track patient’s blood pressure. Portable wireless device that enables monitoring of user’s condition in clinical setting or off-site locations has its potential in improving health care with real-time monitoring and convenience. However, there are also potential risks if misused or misinterpreted. In 2015, the US Food and Drug Administration updated the 2013 document titled “Mobile Medical Applications: Guidance for Industry and Food and Drug Administration Staff” [37]. According to the document, if a blood pressure monitoring device was used for self-management of hypertension without providing specific treatment or treatment suggestion, it would not be regulated under the Federal Food, Drug, and Cosmetic Act [38]. However, if this device was intended for patient-specific analysis and providing patient-specific diagnosis or treatment recommendation, which seemed to be close to the next step of mHealth, it should be treated as a regulated medical device because it could pose risk to the general public. Moreover, reliability, validity, and preparation of regulatory pathway in mobile medical devices should be considered early during app design phase to ensure patient safety [39]. In addition, data security, communication between patients and health care providers, and usability should be discussed in a collaborative way between several shareholders of mobile medical apps.

### Limitations

Although more than half the study participants enrolled in this trial were among the top quartile of eligible participants with respect to health care utilization, study participants exhibited relatively high levels of baseline medication adherence and PAM as described previously. The high baseline PAM levels among participants in our study prevented us from investigating the relationship between PAM, wireless self-monitoring, and study outcomes in individuals with low PAM scores.

In addition, the study participants were highly educated, were non-Hispanic white, and a female-dominant population compared with a prevalence study in the United States using National Health and Nutrition Examination Survey [40]. Baseline controlled hypertension group was 56% in self-monitoring group and 33% in control group, which could reduce the effect of wireless self-monitoring program. These factors limit generalizability of this study. Further research will be needed especially in uncontrolled hypertensive population.

Furthermore, a formal evaluation of the engagement in monitoring activities was not included, although we tried to look at the trends of monitoring frequency and mean systolic and diastolic blood pressure levels in a subset of participants. Over the 24 weeks of the study period, more than half of the participants were actively engaged in the self-monitoring program with recommended frequency, which was different in results of decreased adherence to protocol over time for a weight-monitoring app [41]. This might be partly due to the different perspective in weight monitoring and blood pressure monitoring, where blood pressure was considered a more significant burden to patient’s health. In addition, automatic reminders and direct care from nursing staff of the disease management program could affect our usage over time.

Identifying the individual characteristics of mHealth self-monitoring programs that influence behavior will lead to improved systems. Finally, as study participants only participated for 6 months, further studies will be required to evaluate long-term benefit.

### Conclusions

We discovered that a wireless self-monitoring program in hypertensive patients strengthened the relationship between positive changes in PAM and health behaviors and blood pressure control.

Adoption of wireless self-monitoring programs has the potential to enhance strategies for the management of hypertension. Further research should seek to identify individuals who may receive the most benefit from wireless self-monitoring programs.

## Appendix

Multimedia Appendix 1 Number of participants and mean systolic and diastolic blood pressure levels over time.

## Abbreviations

GLTEQ: Godin Leisure-Time Exercise Questionnaire
IQR: interquartile range
JNC 8: Eighth Joint National Committee
mHealth: mobile health
Morisky MAS: Morisky Medication Adherence Scale 8 item
PAM: Patient Activation Measure
