# Supplementary figures and images for: The Influence of Wireless Self-Monitoring Program on the Relationship Between Patient Activation and Health Behaviors, Medication Adherence, and Blood Pressure Levels in Hypertensive Patients: A Substudy of a Randomized Controlled Trial
Source: J Med Internet Res. 2016 Jun 22;18(6):e116. doi: 10.2196/jmir.5429 (PMC4935792; doi:10.2196/jmir.5429)

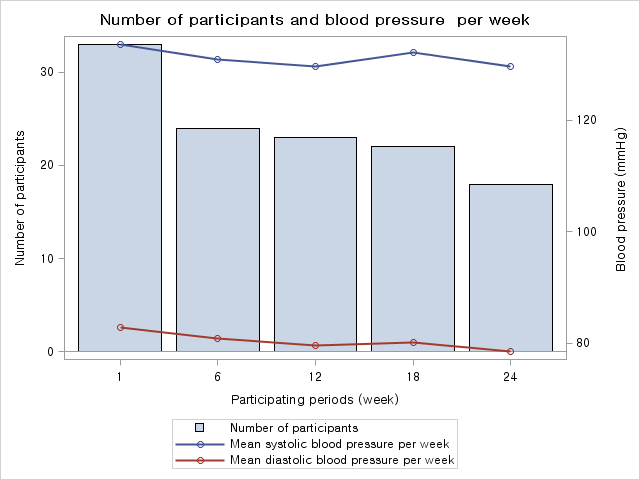

Supplement: Multimedia Appendix 1 [file jmir_v18i6e116_app1.png]
